# Endoplasmic Reticulum Stress Contributes to Ventilator-Induced Diaphragm Atrophy and Weakness in Rats

**DOI:** 10.3389/fphys.2022.897559

**Published:** 2022-06-27

**Authors:** Shaoping Li, Guanguan Luo, Rong Zeng, Lian Lin, Xingnan Zou, Yu Yan, Haoli Ma, Jian Xia, Yan Zhao, Xianlong Zhou

**Affiliations:** Emergency Center, Hubei Clinical Research Center for Emergency and Resuscitation, Zhongnan Hospital of Wuhan University, Wuhan, China

**Keywords:** mechanical ventilation, diaphragm atrophy, diaphragm weakness, oxidative stress, endoplasmic reticulum stress

## Abstract

**Background:** Accumulating evidence indicates that endoplasmic reticulum (ER) stress plays a critical role in the regulation of skeletal muscle mass. In recent years, much attention has been given to ventilator-induced diaphragm dysfunction (VIDD) because it strongly impacts the outcomes of critically ill patients. Current evidence suggests that the enhancement of oxidative stress is essential for the development of VIDD, but there are no data on the effects of ER stress on this pathological process.

**Methods:** VIDD was induced by volume-controlled mechanical ventilation (MV) for 12 h; Spontaneous breathing (SB, for 12 h) rats were used as controls. The ER stress inhibitor 4-phenylbutyrate (4-PBA), the antioxidant N-acetylcysteine (NAC), and the ER stress inducer tunicamycin (TUN) were given before the onset of MV or SB. Diaphragm function, oxidative stress, and ER stress in the diaphragms were measured at the end of the experiments.

**Results:** ER stress was markedly increased in diaphragms relative to that in SB after 12 h of MV (all *p* < 0.001). Inhibition of ER stress by 4-PBA downregulated the expression levels of proteolysis-related genes in skeletal muscle, including *Atrogin*-1 and *MuRF*-1, reduced myofiber atrophy, and improved diaphragm force-generating capacity in rats subjected to MV (all *p* < 0.01). In addition, mitochondrial reactive oxygen species (ROS) production and protein level of 4-HNE (4-hydroxynonenal) were decreased upon 4-PBA treatment in rats during MV (all *p* < 0.01). Interestingly, the 4-PBA treatment also markedly increased the expression of peroxisome proliferator-activated receptor-gamma co-activator-1alpha (PGC-1α) (*p* < 0.01), a master regulator for mitochondrial function and a strong antioxidant. However, the antioxidant NAC failed to reduce ER stress in the diaphragm during MV (*p >* 0.05). Finally, ER stress inducer TUN largely compromised diaphragm dysfunction in the absence of oxidative stress (all *p* < 0.01).

**Conclusion:** ER stress is induced by MV and the inhibition of ER stress alleviates oxidative stress in the diaphragm during MV. In addition, ER stress is responsible for diaphragm dysfunction in the absence of oxidative stress. Therefore, the inhibition of ER stress may be another promising therapeutic approach for the treatment of VIDD.

## Introduction

Mechanical ventilation (MV) is one of the most commonly used life-support techniques with many indications. Unfortunately, this technique carries a high risk of complications, which leads to difficult weaning, a prolonged hospital stay, and increased mortality. It has been demonstrated that diaphragm dysfunction strongly impacts outcomes of mechanically ventilated patients (Goligher et al., 2018), and much attention has been given to ventilator-induced diaphragm dysfunction (VIDD) in recent years. VIDD is a major complication of MV that refers to diaphragm atrophy and force loss (Moroz et al., 2019). Laboratory investigations have suggested that overproduction of reactive oxygen species (ROS) induces ubiquitin-mediated protein degradation in the diaphragm during MV, and numerous antioxidants such as N-acetylcysteine (NAC), MitoTempol, and SS31 can largely relieve the dysfunction of the diaphragm (Shindoh et al., 1990; Zhou et al., 2019; Dridi et al., 2020). Therefore, it has been widely accepted that oxidative stress plays a central role in the pathophysiological process of VIDD. However, the effects of oxidative stress on the development of VIDD have been questioned recently. Some studies have shown that oxidative stress is unnecessary for the development of diaphragm atrophy and contractile dysfunction during MV (van den Berg et al., 2017; Breuer et al., 2018). In addition, antioxidants can not completely ameliorate VIDD, even when given at the very beginning of MV.

Internal and external factors that compromise normal endoplasmic reticulum (ER) function cause accumulation of unfolded or misfolded proteins, which ultimately leads to ER stress (Zhao and Ackerman, 2006). To alleviate stress and restore homeostasis, the unfolded protein response (UPR) is activated in ER, which is mediated by three ER transmembrane sensors: protein kinase R-like endoplasmic reticulum kinase (PERK), inositol-requiring protein 1 (IRE1), and activating transcription factor 6 (ATF6) (Hetz, 2012). Skeletal muscles, including the diaphragm, contain an extensive ER network. It has been demonstrated that ER stress is widely involved in skeletal muscle-related diseases (Bohnert et al., 2018). Induction of the UPR by an appropriate level of ER stress can regulate the metabolism and formation of skeletal muscle by maintaining Ca^2+^ balance and promoting protein folding to improve muscle contraction function (Gallot and Bohnert, 2021). Conversely, chronic ER stress-induced UPR has been shown to be involved in the activation of proteolytic pathway, inhibition of protein synthesis, regulation of skeletal muscle mass and metabolic function (Afroze and Kumar, 2019). Although ER stress is commonly observed in different types of skeletal muscle diseases, little data is available regarding the role of ER stress in diaphragm muscle atrophy and weakness (Bohnert et al., 2018; Afroze and Kumar, 2019; Gallot and Bohnert, 2021). Recently, it has been reported that the presence of ER stress is closely related to diaphragm atrophy and weakness in septic animals (Jiao et al., 2017). Previous studies have demonstrated that sepsis-related diaphragm dysfunction and VIDD shared many key molecular mechanisms, such as oxidative stress and the overexpression of cytokines (Maes et al., 2014; Dridi et al., 2020). In addition, our RNA-seq analysis uncovered that ER stress-related genes is markedly upregulated in the diaphragm after 12 h of MV (Liu et al., 2021a). These findings indicated that ER stress is probably involved in the development of VIDD. However, whether and how ER stress contributes to VIDD has not yet been studied.

To elucidate the role of ER stress in the development of VIDD, we administered 4-phenylbutyrate (4-PBA, an ER stress inhibitor) to adult male Wistar rats subjected to MV and spontaneous breathing (SB). Rats subjected to SB and treated with 4-PBA exhibited more significant protein degradation, diaphragm atrophy, and contractile dysfunction than the untreated SB group. In contrast, 4-PBA largely attenuated diaphragm atrophy and weakness in rats subjected to MV. Next, the relationship between oxidative stress and ER stress in the pathogenesis of VIDD was determined. Our data suggested that ER stress promoted oxidative stress, whereas oxidative stress had little impact on the occurrence of ER stress in the diaphragm during MV. Importantly, our data demonstrated that induction of ER stress by tunicamycin induced apparent diaphragm dysfunction in the absence of oxidative stress in a rat model of VIDD.

## Methods and Materials

### Animals

Adult male Wistar rats weighing 400–450 g were purchased from Charles River Laboratories (Beijing, China). The rats were kept in cages under controlled conditions on a 12:12 light-dark cycle. Water and food were provided ad libitum. All procedures were performed following the Institutional Guidelines of Laboratory Animal Use and Care. The Bio-Safety Level III Animal Laboratory of Wuhan University approved the animal experiments (AUP: SQ20200029).

### Study Design

Study 1: To determine the roles of ER stress in VIDD, animals were randomly assigned into four groups: 1) the spontaneous breathing (SB) without any drug treatment group (*n* = 6), in which animals were tracheostomized and kept for 12 h under anesthesia after the operation (see details in the Mechanical Ventilation section); 2) SB with 4–PBA treatment (SB + 4-PBA) group (*n* = 6), in which animals underwent tracheotomy, received a single intraperitoneal injection of 4-PBA (100 mg⋅kg^−1^, P21005, Sigma-Aldrich, St. Louis, MO, United States), and were then allowed to breathe spontaneously for 12 h under anesthesia; 3) the MV group (*n* = 6), in which animals were mechanically ventilated for 12 h (VT: 5 ml⋅kg^−1^ body weight, PEEP: 8 cm H_2_O) under continuous anesthesia without any additional treatment; and 4) the MV + 4-PBA group (*n* = 6), in which animals were mechanically ventilated for 12 h after an intraperitoneal injection of 4-PBA (100 mg⋅kg^−1^) prior to the onset of MV. The dose of 4-PBA used in the present study was determined based on our experience and a previous report (Bhardwaj et al., 2019).

Study 2: This experiment was designed to determine the relationship between oxidative stress and ER stress in the diaphragm during MV. Animals were randomly assigned to 4 groups: 1) the MV group (*n* = 6), in which animals were mechanically ventilated for 12 h; 2) the MV + NAC group (*n* = 6), in which animals were mechanically ventilated and treated with the antioxidant NAC (A7250, Sigma-Aldrich, St. Louis, MO, United States) *via* a single intraperitoneal injection at a dose of 200 mg⋅kg^−1^ (dissolved in phosphate-buffered saline) prior to the onset of MV; 3) the MV + TUN group (*n* = 6), in which animals were mechanically ventilated and treated with the ER stress-inducing agent TUN (ab120296, Cambridge, MA, United States) *via* a single intraperitoneal injection at a dose of 5 mg⋅kg^−1^ prior to the onset of MV; and 4) the MV + NAC + TUN group (*n* = 6), in which animals were subjected to MV and received both NAC (200 mg⋅kg^−1^) and TUN (5 mg⋅kg^−1^) prior to the onset of ventilation. The dosages of NAC and TUN were determined based on the results of our pilot study and previous reports (Gao et al., 2020; Coşkun et al., 2021).

**FIGURE 1:**
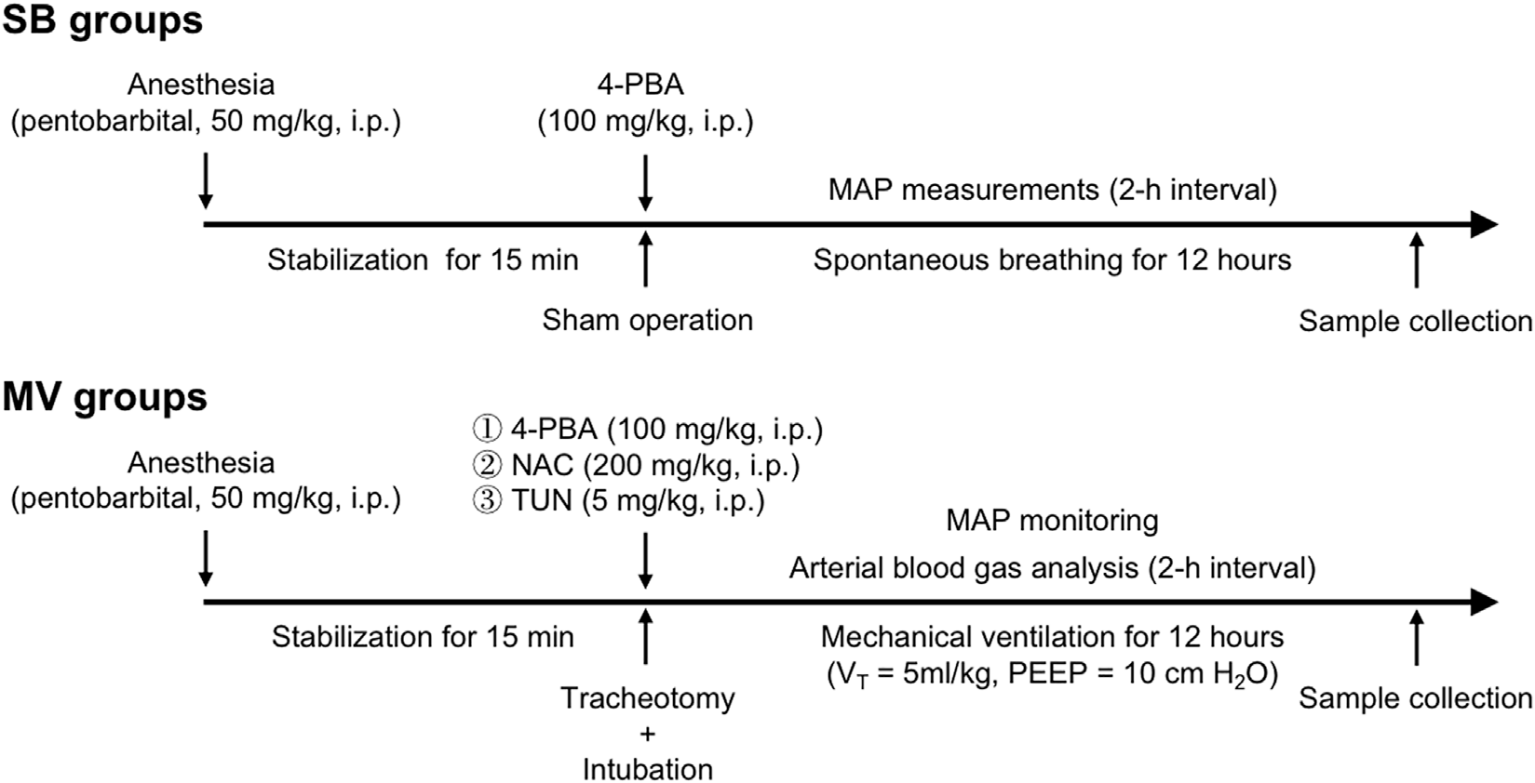
Study design. Animals were randomly assigned to the following groups: the spontaneous breathing (SB), SB + 4-phenylbutyrate (4-PBA, ER stress inhibitor), mechanical ventilation (MV), MV + 4-PBA, MV + tunicamycin (TUN, an ER stress inducer), MV + N-acetylcysteine (NAC) and MV + NAC + TUN groups. n = 6 per group. A single injection of 4-PBA, TUN and/or NAC was given just after the sham operation in the SB groups or prior to the onset of ventilation in the MV groups.

At the end of the experiment, the animals were sacrificed under anesthesia for sample collection. The extensor digitorum longus (EDL) muscle was collected to assess ER stress in hindlimb muscles. The experimental protocol is presented in Figure 1.

### Mechanical Ventilation

A rat MV model was established as described previously (Zhou et al., 2019). Briefly, rats were tracheostomized and ventilated using a small animal ventilator (VentElite, Harvard Apparatus; Cambridge, MA, United States) under anesthesia with sodium pentobarbital (50 mg⋅kg^−1^, i. p.). The breathing air was humidified and enriched with oxygen if necessary. The respiration rate (RR) and oxygen supply were adjusted to maintain a PaO_2_ between 60 and 100 mmHg and a PaCO_2_ between 35 and 45 mmHg during the experiments. The mean arterial pressure (MAP) was monitored using tail-cuff plethysmography (BP-2010, Softron, Japan). The right jugular vein was infused with normal saline at a rate of 1 ml⋅kg^−1^⋅h^−1^ and pentobarbital sodium at a rate of 10 mg⋅kg^−1^⋅h^−1^ to maintain anesthesia. The infusion rate was adjusted to maintain a MAP value above 80 mmHg during MV.

### Blood Analysis

Arterial blood gas analysis (e.g., pH, PaO_2_, and PaCO_2_) and serum lactate levels were determined using a handhold analyzer at a 2-h interval during MV (i-STAT1Analyzer, Abbott, Kyoto, Japan). At the end of the experiment, arterial blood samples were collected for blood cell counts (Pentra MS CRP, HORIBA Medical, Kyoto, Japan). The arterial blood gases analysis was not performed in SB animals.

### Measurement of Diaphragm Force-Generating Capacity

Strips from the right costal diaphragm were removed prior to sacrifice by perfusion. A diaphragmatic muscle strip (approximately 1 cm in length) from each rat was rapidly mounted in a tissue chamber containing Krebs-Henseleit (K-H) solution comprised of the following: 118 mM NaCl, 4.7 mM KCl, 1.2 mM MgSO_4_, 1.1 mM KH_2_PO_4_, 25 mM NaHCO_3_, 2.4 mM CaCl_2_, and 5.5 mM glucose. The K-H solution was bubbled with a mixture of 95% O_2_ + 5% CO_2_ and maintained at 27°C and pH 7.4. Muscle extremities were clamped with spring clips and attached to an electromagnetic force transducer. After 15 min of stabilization, muscle contractile properties were determined as we previously described (Zhou et al., 2019). Force-frequency curves were generated to describe the diaphragm muscle forces.

### Real-Time Quantitative Polymerase Chain Reaction

Real-time quantitative polymerase chain reactions (RT-qPCR) were performed following a standard protocol. Briefly, 30 mg of diaphragm tissue was homogenized for total RNA extraction. Then, the extracted RNA was reverse transcribed using a Revert Aid First Strand cDNA Synthesis kit (Invitrogen, Carlsbad, CA, United States). RT-qPCR for ER stress markers including *GRP78*, C/EBP homologous protein (*CHOP*), and *ATF6*, proteolysis-related genes including atrophy gene-1 (*Atrogin-1*) and muscle RING finger 1 (*MuRF-1*), and the antioxidant *PGC-1α* were examined using the following primer sets:

*GRP78*-F: 5'-CAT​CAC​GCC​GTC​CTA​TGT​CG-3';

*GRP78-R*: 5'-CGT​CAA​AGA​CCG​TGT​TCT​CG-3';

*CHOP*-F: 5'-CTG​GAA​GCC​TGG​TAT​GAG​GAT-3';

*CHOP*-R: 5'-CAG​GGT​CAA​GAG​TAG​TGA​AGG​T-3';

*ATF6*-F: 5'-GAC​TCA​CCC​ATC​CGA​GTT​GTG-3';

*ATF6*-R: 5'-CTC​CCA​GTC​TTC​ATC​TGG​TCC-3';

*MuRF-1*-F: 5'-GTG​TGA​GGT​GCC​TAC​TTG​CTC-3';

*MuRF-1*-R: 5'-GCT​CAG​TCT​TCT​GTC​CTT​GGA-3';

*Atrogin-*1-F: 5'-CAG​CTT​CGT​GAG​CGA​CCT​C-3';

*Atrogin-*1-R: 5'-GGC​AGT​CGA​GAA​GTC​CAG​TC-3';

*GAPDH*-F: 5'- AGG​TCG​GTG​TGA​ACG​GAT​TTG-3';

*GAPDH*-R: 5'-G TGT​AGA​CCA​TGT​AGT​TGA​GGT​CA-3';

*PGC-1α*-F: 5'-TAT​GGA​GTG​ACA​TAG​AGT​GTG​CT-3';

*PGC-1α*-R: 5'-CCA​CTT​CAA​TCC​ACC​CAG​AAA​G-3'.

mRNA expression levels were quantified using the 2-ΔΔCq method.

### Western Blot Analysis

The protein levels of markers of ER stress (GRP78, CHOP, and ATF6), protein degradation (Atrogin-1 and MuRF-1), and lipid oxidation (4-HNE) were measured by immunoblotting. In detail, 50 mg of diaphragm tissue was immersed in 800 μL RIPA lysis buffer (50 mM Tris (pH 7.4), 150 mM NaCl, 1% NP-40, 0.5% sodium deoxycholate, and 0.1% SDS) containing a protease inhibitor cocktail (P0013C, Beyotime, Shanghai, China) and homogenized on ice. Protein concentrations of the samples were determined by a BCA kit (P0012S, Beyotime, Shanghai, China) following the manufacturer’s manual. Then, the samples were boiled after the addition of an appropriate amount of a loading buffer. Approximately 20 μg of each sample was separated by 10% SDS–PAGE and transferred onto a PVDF membrane (Millipore, Bedford, MA, United States). The membranes were incubated with primary antibodies, including Atrogin-1 (ab168372, 1:1000), MuRF-1 (ab172479, 1:1000), CHOP (ab11419, 1:1000), GRP78 (ab21685, 1:1000), ATF6 (ab37149, 1:1000), 4-HNE (ab46545, 1:1000), PGC-1ɑ (ab191838, 1:1000), and GAPDH (ab181602, 1:1000) antibodies (Abcam, Cambridge, MA, United States) at 4°C overnight. After washing with Tris-buffered Saline and Tween 20 (TBST), the membranes were further incubated with HRP-labeled secondary antibodies (ba1058, Boster, Wuhan, China). Finally, an enhanced chemiluminescence reagent (P1000, Pulilai, Beijing, China) was added to the surface of the membranes and allowed to react with the protein bands. Images were captured with a luminescence imaging system (Tanon-6200, China) and analyzed by ImageJ software (v1.46 k; National Institute of Health, Bethesda, MA). GAPDH was used as a loading control.

### Mitochondrial ROS Production

Mitochondrial ROS production in the diaphragm was determined using the Amplex Red™ reagent (Life Technologies, CA, United States) as previously described (Kavazis et al., 2009). Briefly, diaphragm muscle fiber bundles were sequentially incubated with succinate, creatine kinase, creatine phosphate, and creatine at 37°C. Then, sample fluorescence was measured at 15 min after incubation, and the fluorescence was normalized to the weight of the dry tissue of controls.

### Measurement of Myofiber Cross-Sectional Areas

Diaphragm tissue samples were embedded in Optimal Cutting Temperature (OCT), placed in liquid nitrogen, and stored in a refrigerator at −80°C. Then, immunofluorescence staining was performed using frozen tissue samples to evaluate the cross-sectional area (CSA) of diaphragmatic myofibers. In detail, NOQ7.5.4D (ab11083, Abcam) and MY-32 antibodies (ab51263, Abcam) were used for the identification of slow and fast myosin heavy chains, respectively. An anti-laminin antibody (ab11575, Abcam) was used to visualize the outline of the myofibers. Images were obtained using an Olympus IX73 microscope (Olympus Co., Japan), and the CSA was calculated with ImageJ software (Fiji) for at least 200 fibers per animal.

### Statistical Analysis

The data are expressed as the mean ± standard deviation (SD). Comparisons between two groups were performed by unpaired Student’s *t*-test. When more than two groups were compared, two-way ANOVA was performed followed by the Tukey honestly significant difference (HSD) post hoc test if appropriate. All statistical analyses were performed using STATA (version 15.1, StataCorp LLC, United States). A two-tailed *p* value less than 0.05 was considered significant. The number of animals per group required to identify significant differences in major parameters was determined based on previous experience with the same model, and no missing values were documented in this study.

## Results

### Ventilator Parameters and the Systemic Response to SB and MV

A total of 45 rats were included. Five rats were excluded before ventilation because of un-controlling arterial bleeding during arterial cannulation. Then 40 rats were randomly assigned into designed groups, and all animals survived until the schedule sacrifice. First, we performed experiments to examine whether MV would cause physiological differences compared to SB. We found no significant differences in respiration rate (RR) or tidal volume (V_T_) between the MV group alone and MV plus drugs treatment groups. Blood gas analysis showed that the arterial pH, PaO_2_, and PaCO_2_ values were similar between the SB and MV groups. Of note, the oxygen supply and respiration rate were adjusted according to the results of the blood gas analysis to maintain arterial PaO_2_, and PaCO_2_ within the normal range. The lactate levels were slightly increased in the MV groups compared with the SB groups, but the differences were not statistically significant (*p* > 0.05). In addition, the cell count of leukocytes and neutrophils indicated no apparent infection in rats subjected to MV. The mean arterial pressure (MAP) in the MV group was significantly lower than that in the SB group at the end of the experiment (98 ± 7 vs. 134 ± 7 mmHg, *p* = 0.0017). The difference in MAP between the MV groups was not significant, and the MAP was maintained above 80 mmHg by normal saline infusion during ventilation. The results are summarized in Table 1.

**TABLE 1:** Ventilator parameters and systemic response.

Group		RR (bpm)	V_T_ (ml)	Arterial pH	PaO_2_ (mmHg)	PaCO_2_ (mmHg)	Lactate (mmol/L)	Erythrocyte (×10^12^/L)	Leukocytes (×10^9^/L)	Neutrophils (×10^9^/L)	Hemoglobin (g/L)	MAP (mmHg)
Study 1	SB	NA	NA	7.41 ± 0.03	88 ± 6	40 ± 2	0.85 ± 0.20	6.92 ± 0.24	6.73 ± 0.54	0.68 ± 0.12	152 ± 10	134 ± 7
	SB+4-PBA	NA	NA	7.41 ± 0.03	90 ± 4	40 ± 3	0.74 ± 0.16	6.93 ± 0.27	6.73 ± 0.51	0.63 ± 0.15	149 ± 9	132 ± 5
	MV	60 ± 2	2.2 ± 0.1	7.39 ± 0.04	96 ± 6	39 ± 2	0.97 ± 0.21	6.93 ± 0.22	6.62 ± 0.53	0.71 ± 0.11	142 ± 3	98 ± 7*
	MV+4-PBA	59 ± 2	2.1 ± 0.1	7.39 ± 0.04	92 ± 6	37 ± 3	1.07 ± 0.13	6.75 ± 0.27	6.78 ± 0.33	0.71 ± 0.29	146 ± 6	100 ± 6*
Study 2	MV	57 ± 3	2.1 ± 0.1	7.40 ± 0.03	93 ± 4	40 ± 3	1.01 ± 0.15	6.82 ± 0.21	6.54 ± 0.29	0.68 ± 0.16	143 ± 5	102 ± 6*
	MV + TUN	59 ± 4	2.2 ± 0.1	7.39 ± 0.03	96 ± 8	36 ± 3	1.00 ± 0.14	6.82 ± 0.25	6.68 ± 0.36	0.68 ± 0.18	147 ± 8	102 ± 9*
	MV + NAC	59 ± 2	2.1 ± 0.1	7.39 ± 0.07	93 ± 9	38 ± 3	1.01 ± 0.18	6.68 ± 0.21	6.64 ± 0.43	0.70 ± 0.17	148 ± 4	99 ± 4*
	MA + NAC + TUN	58 ± 2	2.1 ± 0.1	7.38 ± 0.06	91 ± 7	38 ± 3	1.05 ± 0.17	6.80 ± 0.23	6.60 ± 0.45	0.70 ± 0.14	147 ± 6	104 ± 4*

The data are expressed as the mean ± SD., Differences between groups were compared using ANOVA, followed by the Tukey HSD, *post hoc* test. *n* = 6 per group. RR, respiration rate; V_T_, tidal volume; SB, spontaneous breathing; MV, mechanical ventilation; TUN, tunicamycin, NAC = N-acetylcysteine, NA, not applicable. At the end of the experiments, the MAP, in the MV, groups were significantly lower than that in the SB, groups. However, the MAP, was maintained above 80 mmHg in all MV, groups and no significant differences were detected between MV, groups. *p < 0.001 vs. SB, groups.

### ER Stress is Increased in the Diaphragm but Not the Limb Muscle During MV

**FIGURE 2:**
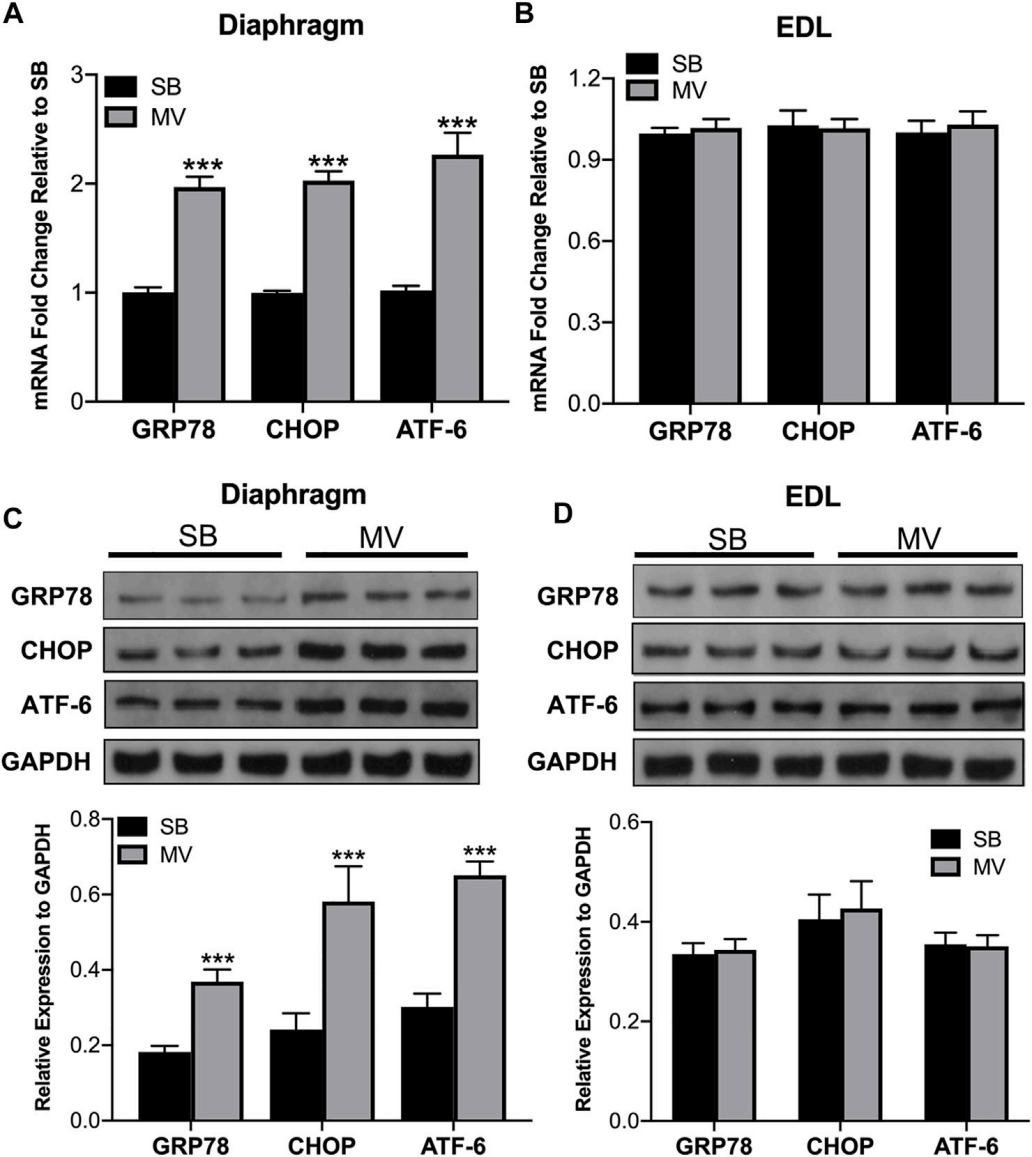
ER stress is induced by MV in the diaphragm. ER stress was measured by quantifying GRP78 CHOP and ATF-6 expression. mRNA transcript levels of ER stress-related genes in the diaphragm **(A)** but not the EDL **(B)** were increased in the MV group compared with the SB group. In addition, immunoblotting revealed marked increases in ER stress-related protein expression in the diaphragm **(C)** but not the EDL **(D)** after MV compared to after SB. ***p < 0.001 versus the SB group. The experiments were repeated three times for each sample (unpaired *t-*test, *n* = 6 per group). MV = mechanical ventilation; SB = spontaneous breathing; EDL = extensor digitorum longus.

To investigate whether ER stress is one of the main reasons that lead to diaphragm dysfunction during MV, we first examined expression levels of several proteins as ER stress biomarkers by RT-qPCR and Western blot analysis. We observed that mRNA levels of the ER stress-related genes, including GRP78, CHOP, and ATF6, exhibited significant upregulation in the diaphragm of rats subjected to MV relative to that from the SB group. (Figure 2A). In contrast, mRNA levels of these genes in limb muscles (the EDL) showed a similar expression in the MV and the SB group (*p* > 0.05) (Figure 2B). Consistently, western blot analysis showed protein levels of GRP78, CHOP, and ATF6 were markedly increased in the MV group as compared with the SB group, whereas no difference was observed in limb muscles between the MV and the SB group (Figures 2C,D). Thus, these results suggest that ER stress in the diaphragm was induced by MV.

### Inhibition of ER Stress Attenuates Diaphragm Atrophy and Weakness During MV

**FIGURE 3:**
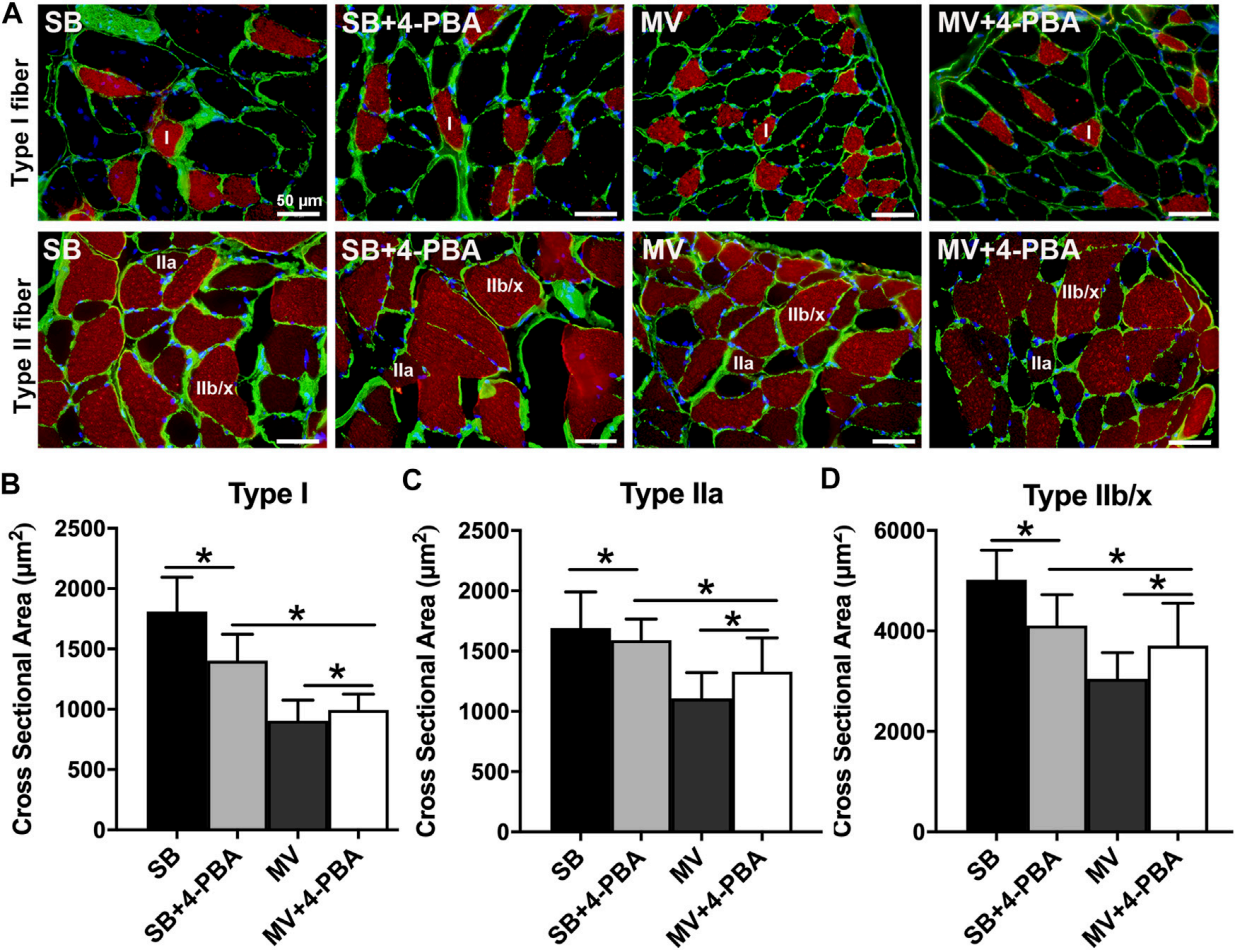
Inhibition of ER stress by 4-PBA reduces diaphragm atrophy during MV. **(A)** Representative immunofluorescence staining images of diaphragm myofibers. **(B–D)** The administration of 4-PBA (ER stress inhibitor) reduced the CSAs of diaphragm myofibers in rats subjected to SB. In contrast, the cross-sectional areas (CSAs) of myofibers in the MV + 4-PBA group were significantly higher than those in the MV group. *p < 0.001 (ANOVA followed by the Tukey HSD test, *n* = 6 per group). MV = mechanical ventilation; SB = spontaneous breathing; 4-PBA = 4-phenylbutyrate.

**FIGURE 4:**
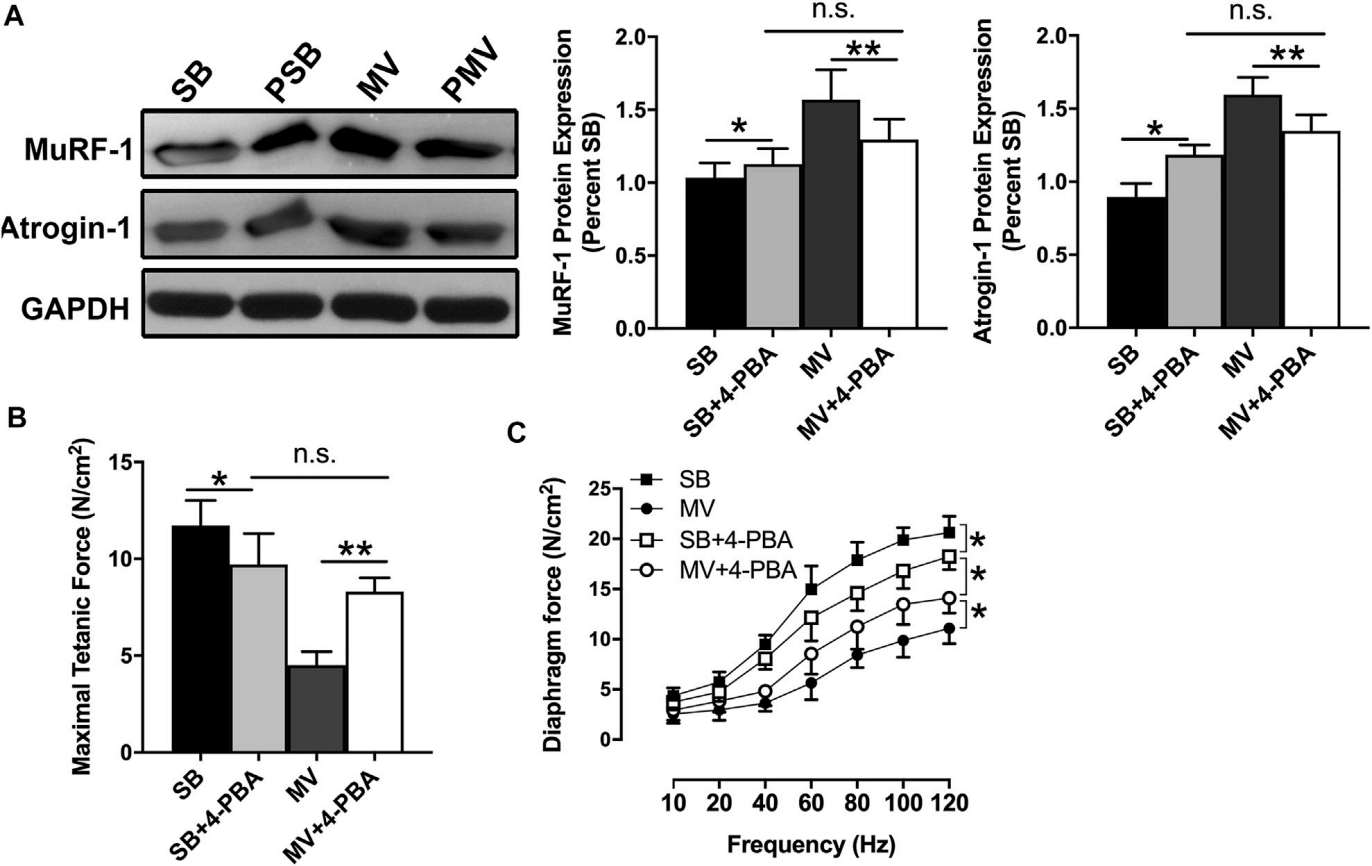
**I**nhibition of ER stress inhibits protein degradation and improves diaphragm contractile capacity during MV. **(A)** Immunoblots for MuRF-1 and Atrogin-1 expressions demonstrated reduced protein degradation in rats undergoing MV after 4-PBA treatment. In addition, diaphragm strips were studied *ex vivo* to evaluate force-generating capacity. The measurements of maximal tetanic forces **(B)** and force-frequency curves **(C)** showed that inhibition of ER stress by 4-PBA resulted in force loss in SB rats and worsened diaphragm weakness in rats undergoing MV. *p < 0.05, **p < 0.01 (ANOVA followed by the Tukey HSD test, *n* = 6 per group). MV = mechanical ventilation; SB = spontaneous breathing; 4-PBA = 4-phenylbutyrate.

Next, we determine whether ER stress is involved in the development of VIDD in rats. First, we compared the CSA of type I, type II, and type IIb/x myofibers of the diaphragm in rats treated with or without 4-PBA, which is a potential ER stress inhibitor, prior to the onset of MV or SB. Immunofluorescence staining experiments were performed by using anti-NOQ7.5.4D, anti-MY-32, and anti-laminin antibodies (Figure 3A). Quantitative analysis confirmed that the CSA of type I, type II, and type IIb/x myofibers were reduced in 4-PBA treated SB groups, whereas they were reversely increased in 4-PBA treated MV groups (all *p* < 0.01). Thus, indicating that myofiber atrophy was alleviated by the administration of 4-PBA (Figures 3B–D). In addition, we examined protein levels of two E3 ubiquitin ligases that are important proteolytic regulators in skeletal muscle. Immunoblots indicated that MuRF-1 and Atrogin-1 were increased upon inhibition of ER stress in SB groups but decreased upon inhibition of ER stress in MV group, which suggested 4-PBA impacts ER stress probably through regulating protein degradation pathway, in which stabilizing E3 ligases in SB groups but degrading them in MV groups (Figure 4A). Moreover, as depicted by the maximal tetanic force and force-frequency curves, the force-generating capacity of the diaphragm was improved in the MV + 4-PBA group compared with the MV group (all *p* < 0.01) (Figures 4B,C). These results demonstrated that the inhibition of ER stress by 4-PBA compromised diaphragm protein degradation, atrophy, and force loss during MV. In contrast, we observed that 4-PBA treatment decreased the CSA of myofibers and force-generating capacity in rats subjected to SB, indicating that 4-PBA could induce diaphragm dysfunction in healthy rats.

### ER Stress Promotes Oxidative Stress by Inhibiting PGC-1α Expression in the Diaphragm During MV

**FIGURE 5:**
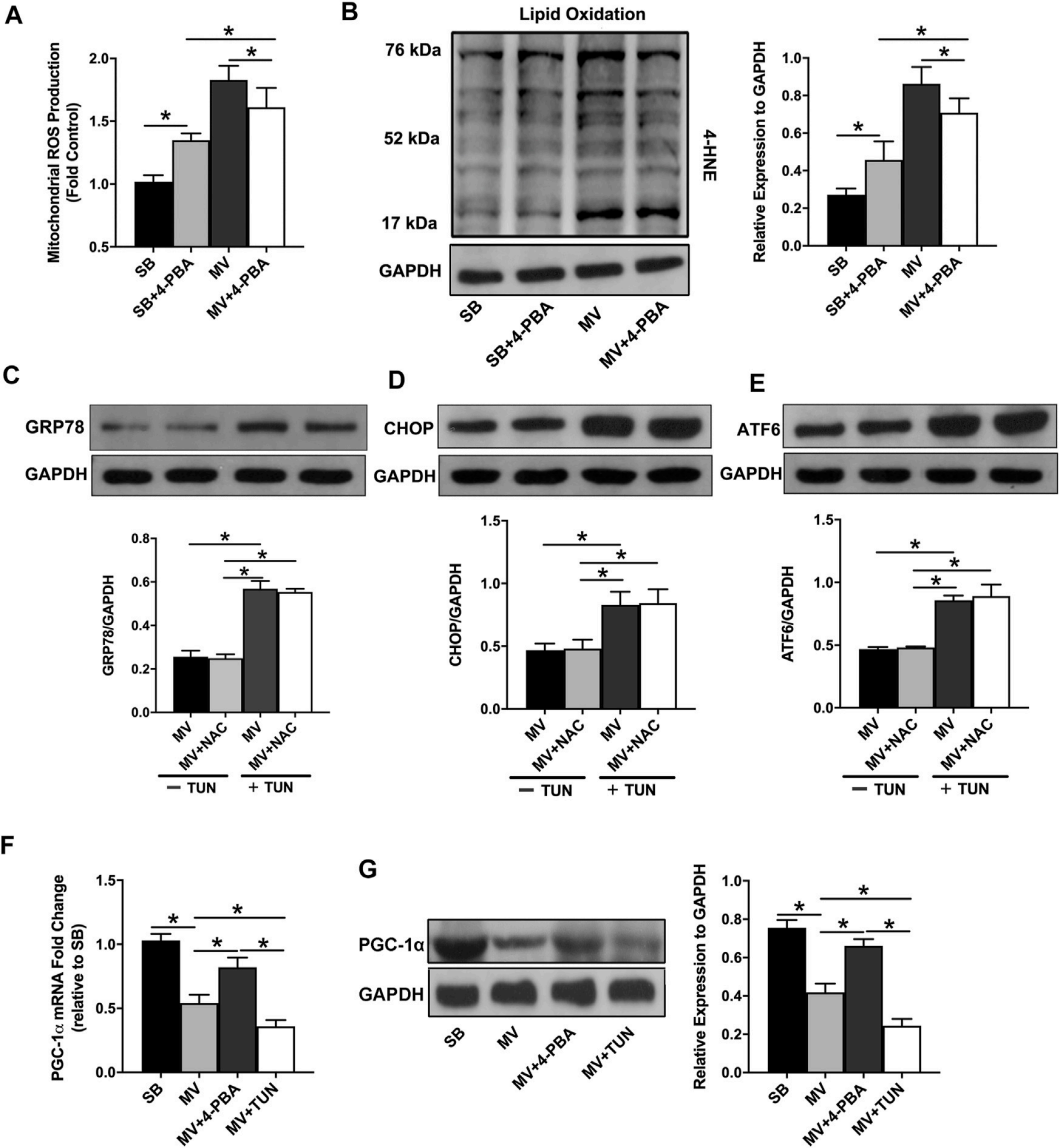
Relationship between ER stress and oxidative stress in the diaphragm during MV. Mitochondrial ROS production **(A)** and lipid oxidation **(B)** were measured at the end of MV. Inhibition of ER stress by 4-PBA decreased oxidative stress in the diaphragm during MV. The protein expression levels of GRP78 **(C)**, CHOP **(D)** and ATF6 **(E)** were measured by immunoblotting to evaluate ER stress levels. Administration of NAC failed to alter ER stress levels in the diaphragm during MV in the absence and presence of the ER stress inducer TUN. RT-PCR **(F)** and western blots **(G)** revealed that PGC-1α mRNA and protein expressions were downregulated by MV in rats. The inhibition of ER stress with 4-PBA upregulated PGC-1α expression in the diaphragm, whereas the induction of ER stress with TUN furtherly decreased its expression in the diaphragm. *p < 0.001 (ANOVA followed by the Tukey HSD test, *n* = 6 per group). ROS = reactive oxygen species, NAC = N-acetylcysteine, MV = mechanical ventilation, 4-PBA = 4-phenylbutyrate, TUN = tunicamycin, 4-HNE = 4-hydroxynonenal.

It has been widely reported that VIDD is associated with oxidative stress and that diaphragm dysfunction can be prevented by the administration of the antioxidant NAC (Shindoh et al., 1990). We wonder whether ER stress could be associated with oxidative stress in the diaphragm during MV. Therefore, we measured the mitochondrial ROS production and the levels of 4-HNE, a reactive aldehydic product of lipid peroxidation to reflect the extent of oxidative stress and mitochondrial dysfunction. Intriguingly, we noticed that inhibition of ER stress by 4-PBA decreased ROS production as well as the 4-HNE level in the diaphragm of MV groups, suggesting that ER stress in MV is positively correlated with oxidative stress (Figures 5A,B). To determine whether oxidative stress also contributes to the occurrence of ER stress, animals received a single injection of NAC prior to the onset of ventilation in the presence or absence of the ER stress-inducing agent TUN. Protein levels of several ER-stress biomarkers during MV were examined by Western blotting. Our results showed that NAC treatment did not affect protein levels of GRP78, CHOP, and ATF6 in the diaphragm in the presence and absence of TUN (*p* > 0.05). However, activation of ER stress by TUN consistently enhanced their expressions (all *p* < 0.05), further verifying the role of ER stress in MV (Figures 5C–E). It has been reported that PGC-1α, the master regulator of mitochondria function, is closely associated with oxidative stress. Thus, mRNA and protein levels of PGC-1α in the diaphragm were compared in SB or MV group with or without 4-PBA or TUN treatment. We observed that diaphragmatic mRNA expression of PGC-1α was markedly decreased after 12 h of MV, while the inhibition of ER stress by addition of 4-PBA, but not activation of ER stress by addition of TUN, conversely promoted PGC-1α expression (all *p* < 0.01, Figure 5F). In line with this result, the PGC-1α protein level was also decreased in the MV group as compared with the SB group, but the use of 4-PBA rescued its protein expression level (*p* < 0.01, Figure 5G). Together, these results strongly suggest that ER stress promotes oxidative stress in the diaphragm very likely by inhibition of PGC-1α expression in the diaphragm.

### ER Stress Induces Diaphragm Atrophy and Weakness in the Absence of Oxidative Stress

**FIGURE 6:**
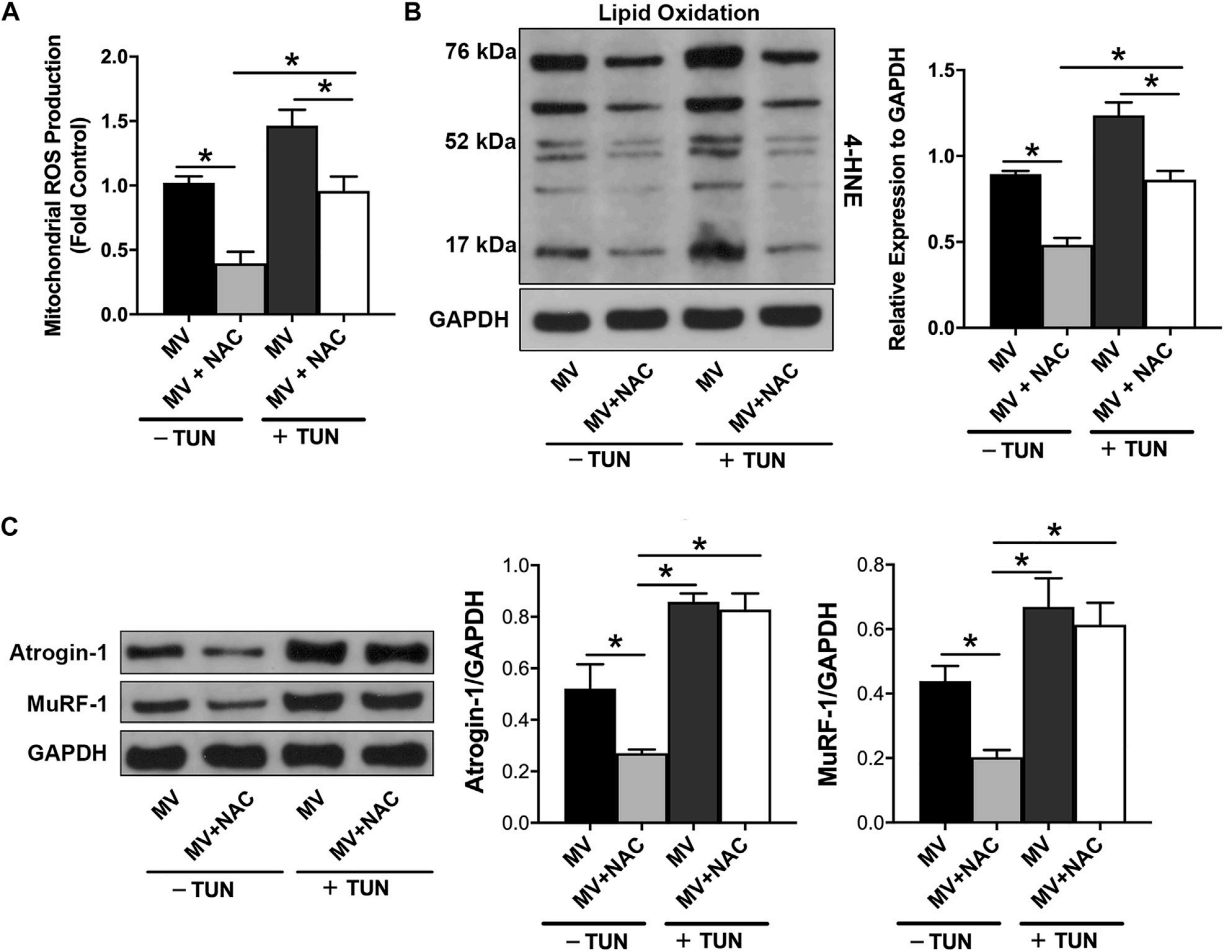
ER stress induces diaphragmatic proteolysis in the absence of oxidative stress during MV. The detection of mitochondrial ROS production **(A)** and analysis of lipid oxidation **(B)** showed that NAC reduced oxidative stress in rats undergoing MV in the absence and presence of TUN. **(C)** Analysis of the protein expression of Atrogin-1 and MuRF-1 suggested that induction of ER stress by TUN enhanced protein degradation in the presence and absence of NAC. *p < 0.001 (ANOVA followed by the Tukey HSD test, n = 6 per group). ROS = reactive oxygen species, NAC = N-acetylcysteine, MV = mechanical ventilation, TUN = tunicamycin, 4-HNE = 4-hydroxynonenal.

**FIGURE 7:**
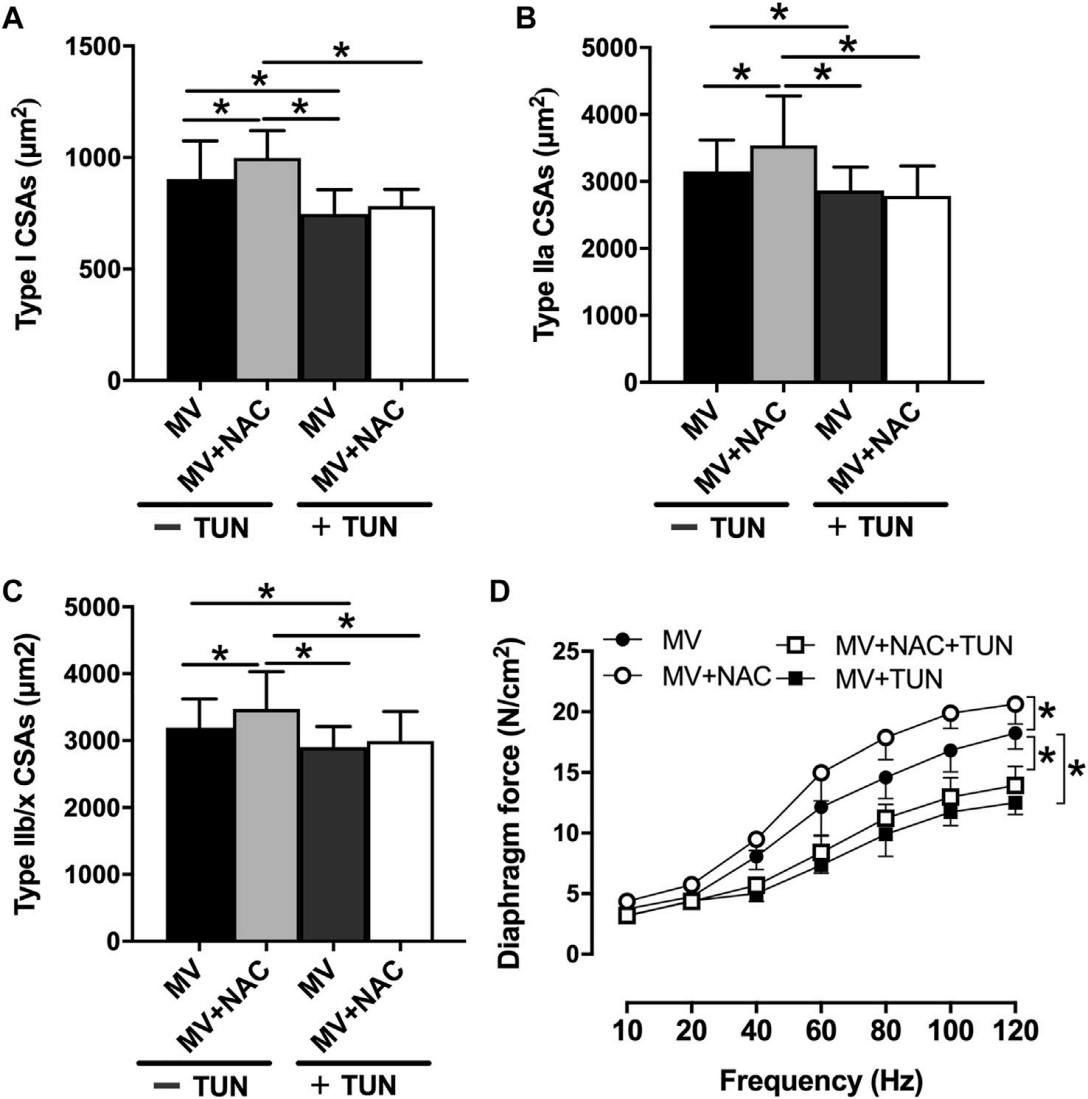
ER stress induces diaphragm dysfunction in the absence of oxidative stress during MV. **(A–C)** Measurement of CSA indicated that induction of ER stress enhanced diaphragm atrophy in rats treated with or without NAC. In addition, diaphragm strips were studied *ex vivo* to evaluate force-generating capacity **(D)**. TUN worsened diaphragm weakness in the presence and absence of oxidative stress in rats in the MV groups. *p < 0.01 (ANOVA followed by the Tukey HSD test, *n* = 6 per group). NAC = N-acetylcysteine, MV = mechanical ventilation, TUN = tunicamycin.

To determine whether ER stress is an independent contributor to the development of diaphragm atrophy and weakness during MV, we analyzed protein degradation, the CSAs of myofibers, and force-generating capacity in animals subjected to MV and treated with NAC and/or TUN. As presented in Figures 6A,B, NAC treatment alleviated mitochondrial ROS production and lipid oxidation in both the absence and presence of TUN-induced ER stress. Immunoblotting for MuRF-1 and Atrogin-1 showed that the administration of NAC reduced protein degradation in the diaphragm during MV, whereas TUN treatment largely promoted diaphragmatic proteolysis in rats undergoing MV. However, the antioxidant NAC failed to alter the protein expression of proteolysis-related genes in the presence of TUN (*p* > 0.05) (Figure 6C). Next, we evaluated the effects of NAC and TUN on diaphragm function during MV. Immunofluorescence staining showed that the CSAs of myofibers in the MV + NAC + TUN group were not statistically significantly lower than those in the MV + TUN group (*p* > 0.05) (Figures 7A–C). The force-frequency curves demonstrated no marked changes in diaphragm forces between the MV + TUN group and the MV + NAC + TUN group (*p* > 0.05) (Figure 7D). Taken together, these results suggested that induction of ER stress worsened diaphragm dysfunction in mechanically ventilated rats. Significantly, ER stress promoted diaphragm dysfunction during MV in the absence of oxidative stress.

## Discussion

To the best of our knowledge, whether ER stress contributes to the development of VIDD has not been addressed. This is also the first study to determine the relationship between ER stress and oxidative stress in the pathogenesis of VIDD. In this study, we mainly found that: 1) ER stress is induced in the diaphragm by MV, and inhibition of ER stress with 4-PBA attenuates VIDD; 2) inhibition of ER stress alleviates oxidative stress in the diaphragm during MV, whereas antioxidants inversely could not affect ER stress; and 3) induction of ER stress exacerbates VIDD in the absence of oxidative stress.

Much attention has been given to VIDD in critically ill patients in the past decade. Pathologically, the development of VIDD is believed to be associated with oxidative stress because overproduction of ROS is commonly observed in the diaphragm during MV, and the administration of antioxidants such as NAC has been proven to protect the diaphragm against VIDD in animals (Shindoh et al., 1990). However, there is little evidence of the effectiveness of antioxidants in the prevention or treatment of VIDD in clinical practice. Conversely, the role of oxidative stress in the development of VIDD has been questioned in recent years Liang et al., (2019) found that oxidative stress is not increased in the diaphragms of newborn lambs with diaphragm atrophy and weakness after MV. In addition, van den Berg and colleagues demonstrated that mechanically ventilated patients admitted to ICUs exhibit diaphragm atrophy and weakness in the absence of mitochondrial dysfunction and oxidative stress (van den Berg et al., 2017). In the present study, we observed that inhibition of oxidative stress with the antioxidant NAC compromised VIDD in rats. NAC was given by injection prior to the onset of MV, and diaphragm atrophy and contractile dysfunction were not completely prevented, which are consistent with previous observations (Shindoh et al., 1990). Furthermore, our results showed that inhibition of ER stress compromised VIDD, whereas activation of ER stress worsened VIDD in the absence of oxidative stress. These evidence suggest that oxidative stress may not play a causative role in the development of atrophy and contractile weakness of the diaphragm during MV and that ER stress may serve as an independent contributor to the pathogenesis of VIDD.

In addition, we are the first to report that inhibition of ER stress with 4-PBA probably reduces diaphragmatic protein degradation. These results align with previous study in septic animals (Jiao et al., 2017). Here, we observed that inhibition of ER stress resulted in decreased expression of two major E3 ligases of the ubiquitin–proteasome proteolysis pathway in the diaphragm. It has been demonstrated that ubiquitin–proteasome-mediated protein degradation is essential for diaphragm atrophy during MV (Tang and Shrager, 2018), and oxidative stress serves as a trigger for the activation of the proteolytic signaling pathway (Moroz et al., 2019). Here, we found that an ER stress inhibitor relieved diaphragmatic oxidative stress in rats; however, the antioxidant NAC failed to ameliorate MV-induced ER stress in the diaphragm. In fact, ER stress leads to the overproduction of ROS, whereas inhibition of oxidative stress does not necessarily inhibit ER stress (Patel et al., 2017). In addition, our previous work (Liu et al., 2021a) and the present study found that the expression of PGC-1α was downregulated by MV in the diaphragm. It has been well documented that PGC-1α is essential for the maintenance of skeletal muscle mass, and its functional deficiency is tightly associated with muscle atrophy (Sandri et al., 2006; Yang et al., 2020). The present study indicated that PGC-1α probably regulates the interactions between ER stress and oxidative stress in the diaphragm during MV. These results may provide a better understanding of oxidative stress and especially ER stress in the diaphragm during MV, which has important clinical significance for formulating treatment strategies for VIDD.

So far, ER stress-induced diaphragm atrophy and contractile dysfunction have not been completely understood. It has been suggested that autophagy is required for VIDD, and inhibition of autophagy reduces the MV-induced production of ROS in the diaphragm (Smuder et al., 2018). In addition, it is now apparent that ER stress is a potent trigger of autophagy (Lee et al., 2015), and our results suggested that ER stress is involved in the development of VIDD. Then, ER stress may lead to diaphragm atrophy by inducing autophagy, which might explain why ER stress induces diaphragm atrophy and weakness in the absence of oxidative stress. In addition, some other signaling pathways, such as the Smad3 (Tang et al., 2017), Janus kinase/signal transducers and activators of transcription (JAK/STAT) (Smith et al., 2014), and forkhead box protein O1 (FOXO1) (Li et al., 2016) pathways, have also been reported to be associated with diaphragm atrophy and weakness during MV. The relationship between ER stress and those signaling pathways has been widely described (Ferreiro et al., 2006; Liu et al., 2018; Vallejo-Gracia et al., 2020; Liu et al., 2021b), but whether the role of ER stress in VIDD is related to these signaling pathways is unclear. Although animal studies have identified the effects of these signaling pathways in the development of VIDD, there is little evidence of the effectiveness of targeted therapies on these signaling pathways in clinical trials. In addition, ER is the major organelle that control the calcium hemostasis in the muscle, and ER stress is always characterized by calcium depletion. Whether calcium depletion is involved in VIDD and its potential mechanisms need to be furtherly investigated. Fortunately, 4-PBA is a Food and Drug Administration (FDA)-approved drug for the management of urea cycle disorders, and its tolerance and safety have been well demonstrated in humans (Mokhtarani et al., 2013; Longo et al., 2021). In addition, it has been suggested that 4-PBA prevents muscle atrophy by modulating ER stress and the ubiquitin-proteasome system (Reddy et al., 2019). These results indicate the potential value of 4-PBA in ER stress management for the prevention or treatment of VIDD.

There are several limitations of this study. First, the experiment was carried out without continuous nutrient infusion or ingestion. So MV was limited to 12 h to avoid the potential hypoglycemia, which maybe has an uncertain effect on the diaphragm. Effects of more prolonged MV (5 days or greater) on the diaphragm function were not examined because animals possibly can not be survived such prolonged MV. The factor of a deteriorating condition which may influence the diaphragmatic ER stress can not be excluded completely. Meanwhile, diseases such as acute respiratory distress syndrome, severe trauma, or burn were not introduced because these factors may have a direct or indirect effect on the diaphragmatic ER stress. Second, whether the inhibition of ER stress by 4-PBA causes diaphragm dysfunction in SB rats has not been investigated in the present study. In addition, we did not determine whether the induction of ER stress by TUN also results in diaphragm atrophy and force loss in SB rats. Third, the effects of ER stress on protein synthesis were not evaluated in this study. Since previous studies have demonstrated that increased protein degradation is essential for diaphragm atrophy than decreased protein synthesis during VIDD (Tang and Shrager, 2018), we only focused on protein degradation in this work. Fourth, blood perfusion in the diaphragm was not determined when the MAP was decreased during MV. Although it has been suggested that diaphragm function and blood perfusion are preserved even during hemorrhagic shock (Carreira et al., 2014), some evidence has shown that the development of VIDD is probably due to a time-dependent reduction in diaphragmatic blood flow and oxygenation (Davis et al., 2012). Fifth, whether oxidative stress at least partly mediates the regulatory effects of ER stress on diaphragm function during MV is unclear. Oxidative stress induction and ER stress inhibition were not combined in rats undergoing MV. Finally, whether PGC-1α mediates the regulatory effects of ER stress on mitochondrial ROS production in the diaphragm during MV is not definitive. The regulation of ER stress together with PGC-1α overexpression or knockdown in the diaphragm during MV should be performed.

## Conclusion

Our study first demonstrated that ER stress is rapidly induced in the diaphragm by MV and is responsible for the development of VIDD in the absence of oxidative stress. Therefore, inhibition of ER stress could be considered as another promising therapeutic approach for VIDD.

## Data Availability

The raw data supporting the conclusions of this article will be made available by the authors, without undue reservation.
